# Improvement of Mouth Functional Disability in Systemic Sclerosis Patients over One Year in a Trial of Fat Transplantation versus Adipose-Derived Stromal Cells

**DOI:** 10.1155/2016/2416192

**Published:** 2016-01-05

**Authors:** Maria Giuseppina Onesti, Paolo Fioramonti, Sara Carella, Pasquale Fino, Cinzia Marchese, Nicolò Scuderi

**Affiliations:** ^1^Dipartimento di Chirurgia “P. Valdoni”, Sapienza University of Rome, Via Giovanni Maria Lancisi 2, 00161 Roma, Italy; ^2^Department of Experimental Medicine, Sapienza University of Rome, Rome, Italy

## Abstract

*Background*. Systemic sclerosis (SSc) is a multisystem disease characterized by cutaneous and visceral fibrosis. Face and mouth changes include telangiectasia, sicca syndrome, and thinning and reduction of mouth width (microcheilia) and opening (microstomia). We applied autologous fat transplantation compared with autologous adipose-derived stromal cells (ADSCs) injection to evaluate the clinical improvement of mouth opening.* Methods*. From February to May 2013 ten consecutive SSc patients were enrolled from the outpatient clinic of Plastic Surgery Department of Sapienza University of Rome. Patients were divided into two groups as follows: 5 patients were treated with fat transplantation and 5 patients received infiltration of ADSCs produced by cell factory of our institution. To value mouth opening, we use the Italian version of Mouth Handicap in Systemic Sclerosis Scale (IvMHISS). Mouth opening was assessed in centimetres (Maximal Mouth Opening, MMO). In order to evaluate compliance and physician and patient satisfaction, we employed a Questionnaire of Satisfaction and the Visual Analogic Scale (VAS) performed before starting study and 1 year after the last treatment.* Results and Conclusion*. We noticed that both procedures obtained significant results but neither one emerged as a first-choice technique. The present clinical experimentation should be regarded as a starting point for further experimental research and clinical trials.

## 1. Introduction

Systemic sclerosis (SSc) or scleroderma is a multisystem disease characterized by cutaneous and visceral fibrosis. After an initial period of induration, dermis and visceral become infiltrated with collagen and become both harder and thicker. It is an autoimmune disease with a prevalence of 2-3 per 10.000 people. The ratio of women to men is 4 to 1, the majority diagnosed between the age of 30 and 50 [1].

Cutaneous manifestations of this disease are very plain and hard to conceal. Limited range of mouth opening, along with other symptoms such as dry mouth, can lead to difficulties with oral hygiene and eating. Facial involvement and oral complications are typical features of SSc, leading to aesthetic changes and impairment of the patient's self-image. The face becomes amimic, cutaneous wrinkles disappear around the mouth, vertical furrows develop, and the nose becomes sharp. Face and mouth changes also include telangiectasia, sicca syndrome, and thinning and reduction of mouth width (microcheilia) and opening (microstomia), also favoured by osteolysis of mandibular angles and by fibrosis of soft tissues.

Sclerosis of the extremities is highly disabling and results in significant dysfunction; the facial symptoms bear cosmetic disfigurement and limit expression, leading to a mask-like stiffness of the face. Currently, therapy is limited and no antifibrotic treatment has proven its efficacy. Recent studies have assessed different biological agents for the treatment of skin thickness as neutralizing antibodies, tyrosine kinase inhibitors, proteins with antifibrotic properties, or proteins that induce immune tolerance, generally well tolerated but not showing significant efficacy [2].

Over the years, autologous fat transplantation has become the first-choice technique to hide cutaneous lesions; this approach, using the patient's own body fat as a natural filler to achieve structural modifications, takes advantage of its abundance and accessibility and avoids complications associated with foreign materials. Elective liposuction for fat transplantation is nowadays considered a safe and well-tolerated procedure [3, 4].

Recently, a stem cell population within the adipose stromal compartment has been identified, termed adipose-derived stromal cells (ADSCs). This stem cell reservoir can be easily obtained from a very small amount of liposuction aspirates (1–5 cc), since it is present in any type of white adipose tissue [5]. Moreover, ADSCs possess the ability to differentiate into various cell types, including adipocytes, chondrocytes, osteoblasts, myocytes, and neurons under specific differentiation conditions. Some studies describe the potential use of ADSCs in treating some autoimmune and inflammatory disorders, such as type I diabetes mellitus, systemic sclerosis and systemic lupus erythematosus, myasthenia gravis, and multiple sclerosis [6–8].

In this project, we applied the procedure of autologous fat transplantation compared with autologous ADSCs injection to evaluate the clinical improvement of mouth opening.

## 2. Materials and Methods

From February to May 2013, ten consecutive SSc patients (8 female and 2 male), fulfilling American College of Rheumatology (ACR) criteria and classified as having diffuse cutaneous scleroderma [9], were enrolled from the outpatient clinic of the Department of Plastic Surgery of Sapienza University of Rome and agreed by a written informed consent to participate in the study, which was approved by our ethics committee (Ref. 1834/25.03.10) and conducted in full accordance with ethical principles, including the World Medical Association Declaration of Helsinki.

These patients had advanced systemic sclerosis-related perioral thickening and mouth opening limitation. The group was homogeneous for age (age range: 20–48 years), disease state, and duration and finally for clinical characteristics.

At each visit, personal, anamnestic, and objective (clinical characteristics) data were collected and recorded, using a written form that was held securely, thus being accessible only to study investigators (Table 1).

Inclusion criteria called for signs of no active disease expressed by increasing size of lesions, appearance of new lesions, and/or clinical signs of inflammation within the last 6 months.

Exclusion criteria were as follows: pregnancy or lactation, any immunomodulating or immunosuppressive therapy within the last 4 weeks and any topical therapy within the last 2 weeks except for the use of emollients, and finally patient's refusal to participate in the study.

Patients were divided into two groups as follows: 5 patients were treated with fat transplantation “group L” (lipofilling) and 5 patients received infiltration of ADSCs produced by cell factory of our institution “group A” (ADSCs). The patients did not receive any remuneration for their inclusion or treatment in this study.

After the first treatment, all patients underwent the same procedure 3 months later. Follow-up was at 1 week, 1 month, and 1 year. During the follow-up, it was possible to compare our obtained results by using fat transplantation with ADSCs infiltration.

### 2.1. Disability Evaluation

We want to describe several parameters used to value disability often experienced by scleroderma patients. Various modalities can be used to measure the extent and severity of skin involvement. The modified Rodnan Skin Score (MRSS), a summation of physical examination ratings over 17 skin sites (fingers, hands, forearms, arms, face, chest abdomen, thighs, lower legs, and feet), has become the standard primary outcome measure of skin involvement during clinical trials and in practice [10].

MRSS is the current gold standard measure of skin disease, but other methods that are more objective, precise, and reproducible have been developed to assess skin involvement. These include skin biopsy, ultrasonography, electronic tonometry, cytometry, and durometry [11–16].

There are also different scales to value quality of life: the Health-Related Quality of Life (HRQoL), the Health Assessment Questionnaire (HAQ), and scleroderma HAQ (sHAQ). This latter is more specific for SSc, as it adds to HAQ 5 visual analogue scales, evaluating Raynaud's phenomenon, digital ulcers, gastrointestinal and lung symptoms, and overall disease severity [17–19].

Hand disability can be studied by specific instruments, such as Cochin Hand Function Scale (CHFS) and Hand Mobility in Scleroderma Scale (HAMIS) [20, 21].

In our study, we decided to examine the following parameters. Skin tightening due to subcutaneous and ligamentous collagen deposit associated with diffuse systemic sclerosis results in a mechanical inability to open the mouth. To value the mouth disability we use the Italian version of Mouth Handicap in Systemic Sclerosis Scale (IvMHISS).

MHISS assessing the handicap with mouth disability in SSc consists of 12 items (each scored 0–4) with a total score range from 0 to 48 (Table 2) [22–27].

Mouth opening was assessed in centimetres (Maximal Mouth Opening, MMO) by measuring the distance between the tips of upper and lower right incisive teeth (mean of two consecutive measurements).

The patients were asked to fill in a questionnaire in which their degree of satisfaction could be expressed by the following ratings: unsatisfied, moderately satisfied, rather satisfied, and very satisfied.

In order to assess* compliance and physician and patient satisfaction* we employed an evaluation system according to the Visual Analogic Scale (VAS) giving a score ranging from 1 up to 10, where 1 indicates no improvement and 10 indicates the maximum possible improvement.

IvMHISS, MMO, Questionnaire of Satisfaction, and VAS were performed before starting study and 1 year after the last treatment.

### 2.2. Case Report 1

**Figure 1:**
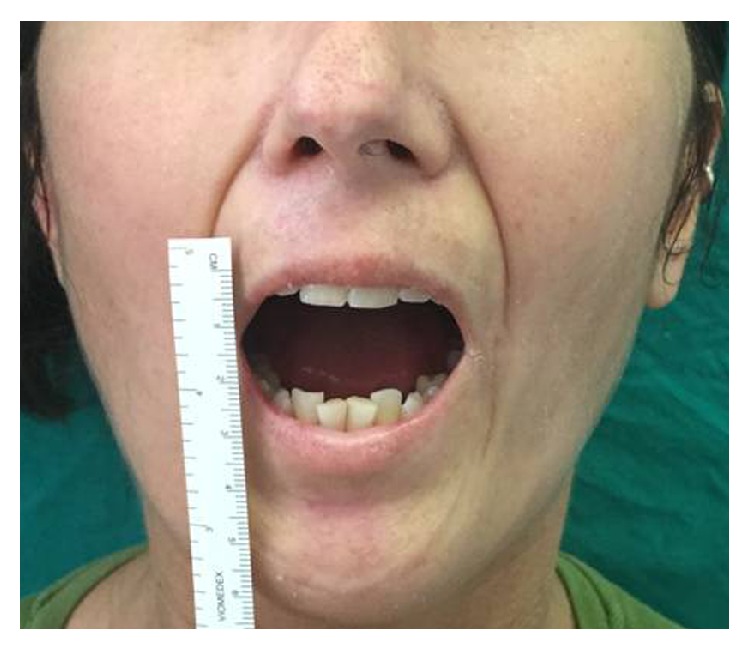
A 39-year-old woman with microstomia before treatment.

**Figure 2:**
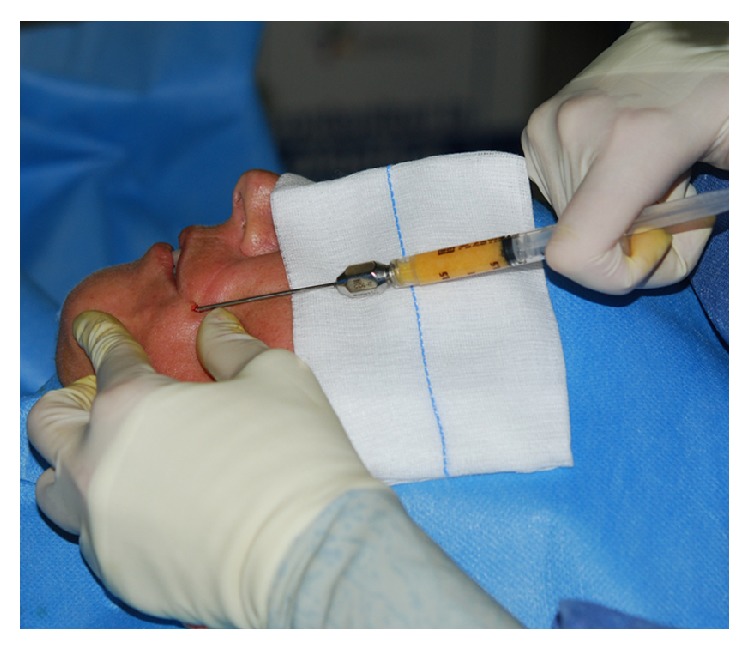
Autologous fat transplantation.

**Figure 3:**
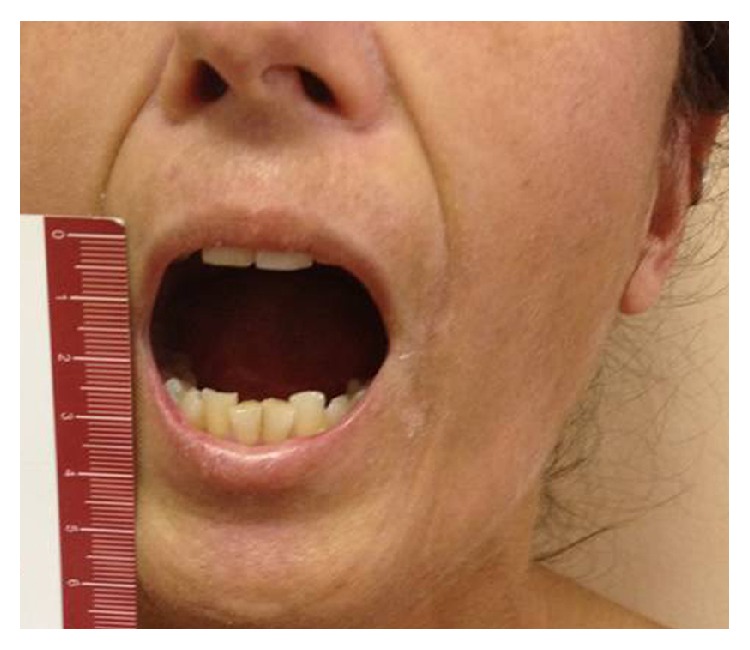
Follow-up at 1 year.

*Patient: A 39-Year-Old Woman*. She first reported the onset of the disease at the age of 25. Disease was characterized by slow but progressive symmetrical skin thickening limited to the fingers (sclerodactyly) and to the face (especially microstomia). She reported that for 18 years the disease was stable (Figure 1). The patient underwent fat transplantation (Figures 2 and 3).

### 2.3. Case Report 2

**Figure 4:**
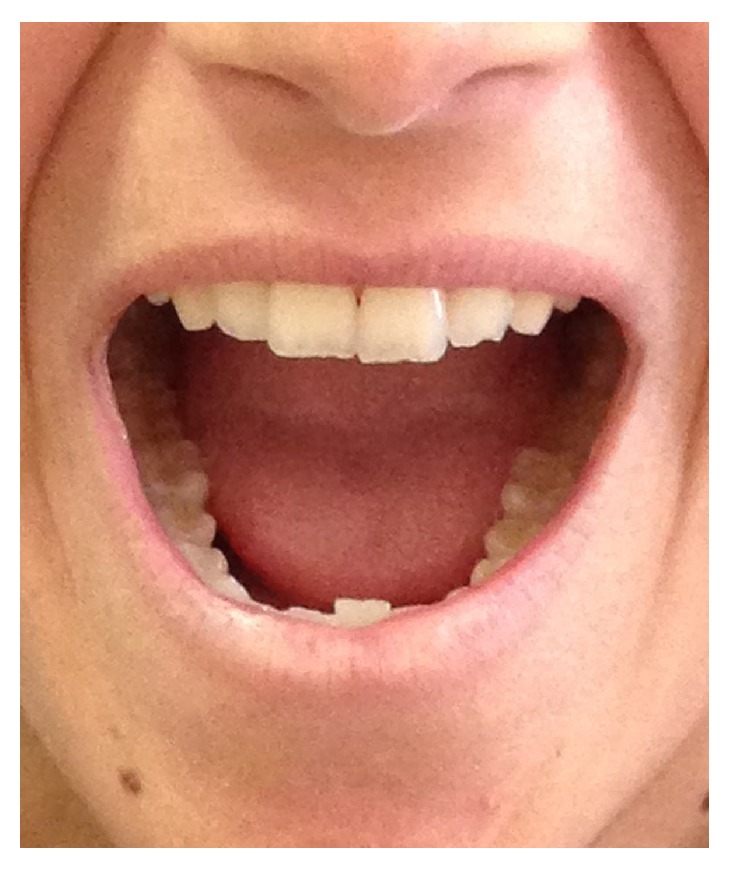
A 38-year-old woman with microstomia before treatment.

**Figure 5:**
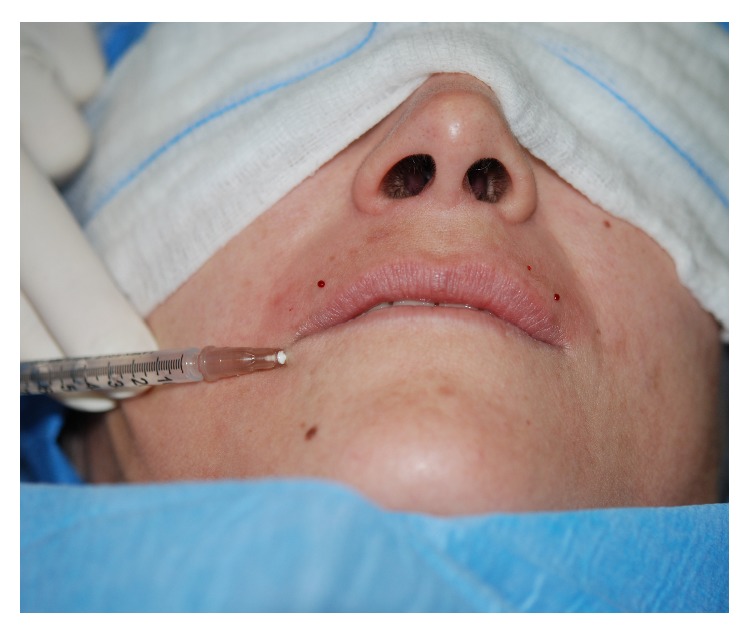
ADSCs injection.

**Figure 6:**
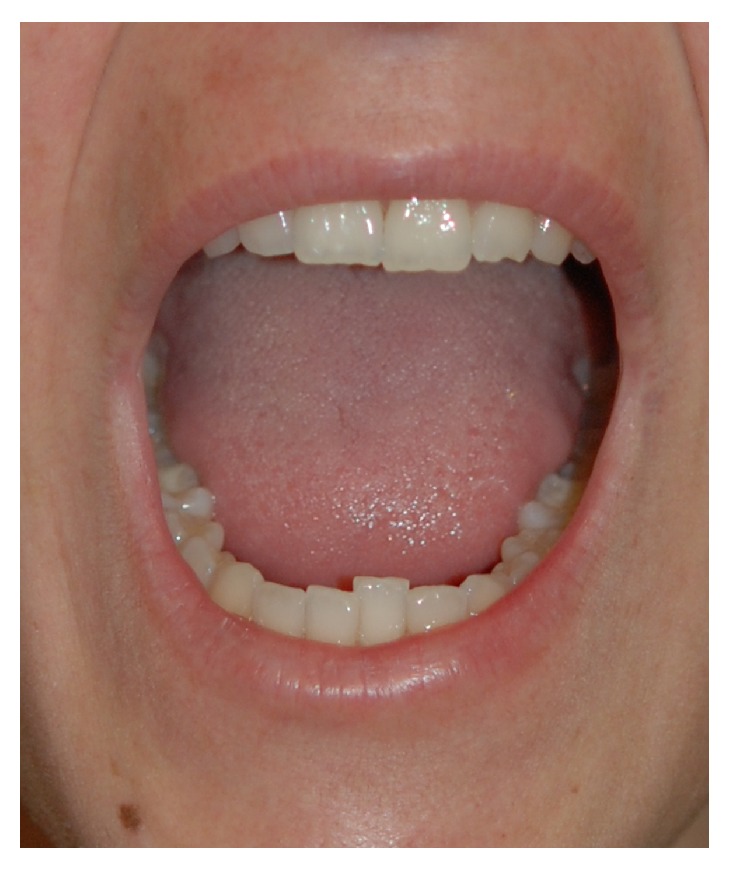
Follow-up at 1 year.

*Patient: A 38-Year-Old Woman*. She reported the disease onset at 28 years of age. Disease was characterized by fast symmetrical skin thickening limited to either the fingers (sclerodactyly) or to the face (especially microstomia). She also reported that for 16 years the disease has not progressed (Figure 4). The patient underwent ADSCs infiltration (Figures 5 and 6).

### 2.4. Technique

#### 2.4.1. Autologous Fat Transplantation Procedure

The periumbilical abdominal region represented the donor site for all patients. After the administration of local modified Klein solution, 1 liter of sodium chloride 0.9%, 20 mL of lidocaine 2%, and 1 mL of epinephrine 1 : 200,000, adipose tissue was harvested using hand-generated suction by means of a one-hole blunt 3 mm cannula attached to a 10 cc Luer-lock syringe. Such nontraumatic low-negative pressure drain method preserves adipocytes intact and viable for transfer [28].

A total amount of 40 mL of lipoaspirate was harvested from the abdomen. Afterward, it was decanted 15 minutes and only the layer containing adipocytes was used for fat injection. The fat infiltration was performed using a blunt injection cannula of 2 mm in diameter. Perioral region was injected using many radiating passages at the subcutaneous level for a total of 16 mL. The cannula was inserted in 4 symmetric sites: 2 located just upon and 2 just below labial commissures. Antibiotics were given to all patients as a precautionary measure.

The selected areas for fat injection were six. Three were at the level of upper perioral region: two at the level of nasolabial fold (injected fat amount: 2 mL for each side) and one at the level of the upper lip (in which we injected 2 mL from the right side of the upper lip to the center and 2 mL from the left side of the upper lip to the center, both at the level of vermilion border). The other three selected areas were in the lower perioral region: two at the level of a line extending from the labial commissure toward mandibular border (injected fat amount: 2 mL for each side) and one at the level of the lower lip (in which we injected 2 mL from the right side of the lower lip to the center and 2 mL from the left side of the lower lip to the center).

#### 2.4.2. ADSCs Isolation, Expansion (Standard Culture Method), and Injection

A total amount of 20 mL of lipoaspirate was harvested from the abdomen with the same technique of fat transplantation. Lipoaspirate was sent to the laboratory for cell cultivation within 1 hour and processed for ADSCs isolation.

Primary cultures of ADSCs derived from each scleroderma patient were expanded following the guidelines of current GMP. On the day of transplantation, cells were detached with 0.5 mM EDTA/0.05% trypsin for 5 min at 37°C and counted. Then, ADSCs were centrifuged at 1,500 rpm for 10 min, washed twice in PBS to remove serum, and finally resuspended in an adequate volume of synthetic stabilized HA solution (a 1.6% solution of synthetic HA, without chemical modifications and with a molecular weight of 1 × 10^3^ KDa, very similar to the endogenous HA) at a standard concentration of 8 × 10^5^ cells/mL. After gentle mixing, the suspension was kept under ambient conditions for 10–15 min to allow cell adherence to the hyaluronan matrix. Homogeneous dispersion of the cells within the gel was ensured by microscopical observation. Then, the cell supplemented HA solution was loaded into an injection syringe and carried to the operating room [29].

Usually after 3 weeks the patient went back to the operating room for the injection of the expanded ADSCs. This procedure did not require anaesthesia; only one patient asked for blunt sedation to calm down. The injection technique relied on preoperative topographic markings. Small aliquots of cell-enriched HA were infiltrated in the chosen areas.

The infiltration was done using 2 mL syringes provided with a 30-gauge 1/2 needle. We employed always 4 mL of hyaluronic acid for each patient, keeping a constant rate of 8 × 10^5^ expanded ADSCs for each mL of HA.

Small aliquots of cell transferred by hyaluronic acid were infiltrated at the subcutaneous level of selected perioral regions: six areas, two in the upper lip and two in the lower lip (two lateral for each lip), plus one area for each opposite mouth corner region.

## 3. Results

All patients treated presented a favourable outcome with improvement in subjective wellness of the skin in the perioral areas.

Both procedures improved the scores of IvMHISS scale at T1 versus T0. A significant score increase was shown in group L (*p* value 0.0234; *t*: 2.7940) and in group A (*p* value 0.0022; *t*: 4.4453); instead there was no statistical significant difference in improvement between groups L and A (*p* value 0.9619; *t*: 0.0485).

**Figure 7:**
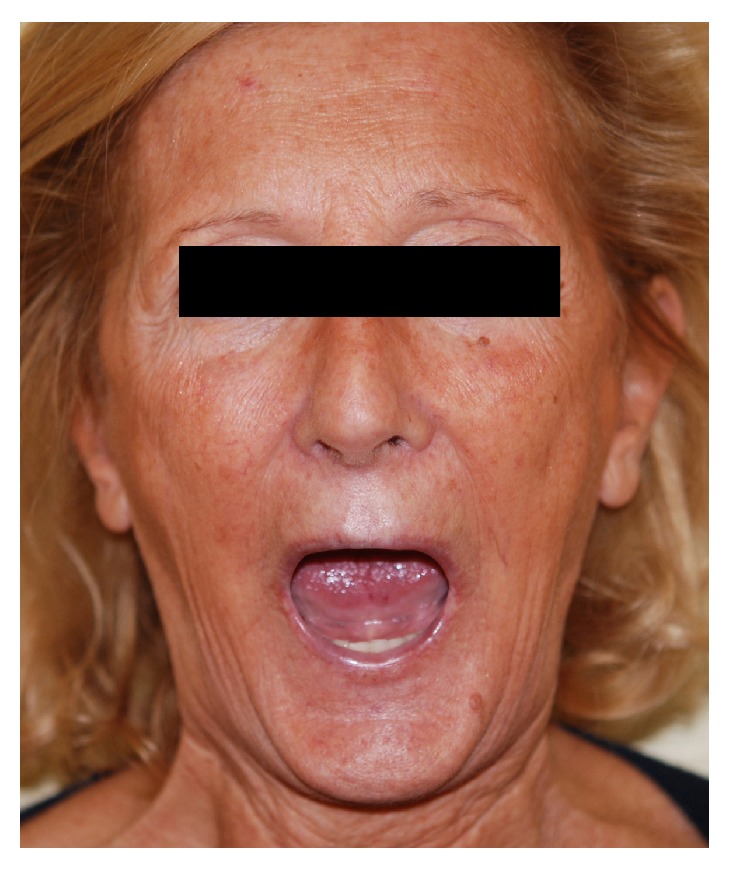
A 48-year-old woman before treatment.

**Figure 8:**
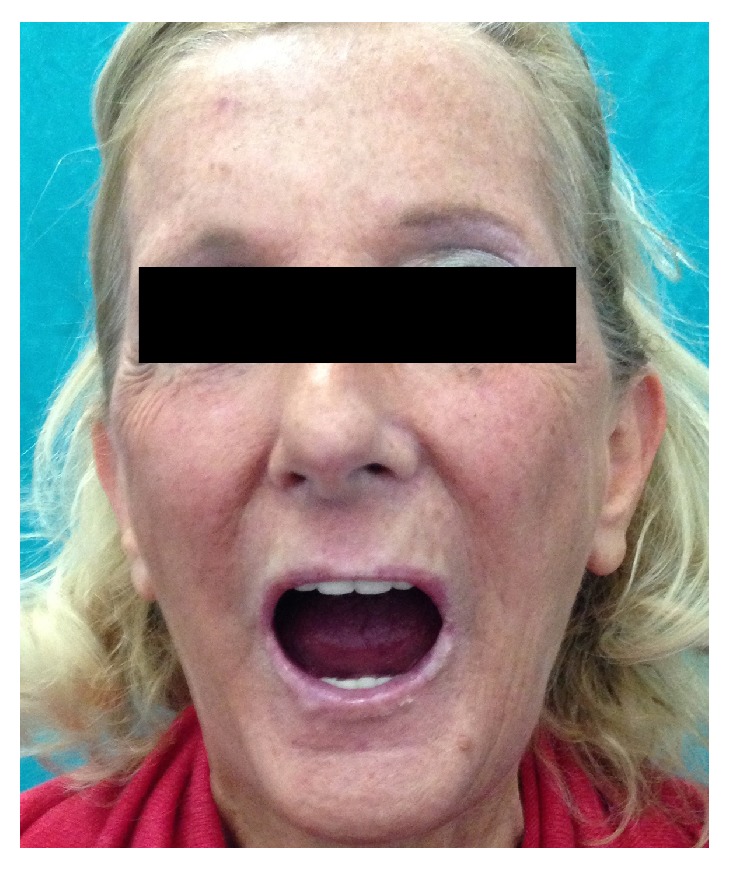
Aesthetic changes in the perioral region after autologous fat transplantation.

Maximal Mouth Opening, assessed as interincisor distance (Figures 7 and 8), was assessed by the same operator at baseline (T0) and after 1-year follow-up (T1). Patients of both groups benefited from the treatments for mouth opening (Table 3). A significant increase of mouth opening was shown in group L (*p* value 0.0171; *t*: 2.9994) and in group A (*p* value 0.0322; *t*: 2.5873); instead the difference of improvement between groups L and A was statistically insignificant (*p* value 0.5833; *t*: 0.5587).

When the patients were asked to express their overall personal opinion on the procedure they had undergone and its effectiveness, 80% (4/5) and 20% (1/5) in group L claimed to be rather satisfied and very satisfied, respectively. In group A, 20% (1/5) and 80% (4/5) claimed to be rather satisfied and very satisfied, respectively.

High values of VAS scale were obtained in both groups as shown in Table 4; instead the difference between groups L and A in terms of improvement was statistically insignificant (*p* value 0.0339; *t*: 2.5560) (i.e., we obtained improvement in groups A and L with both techniques, without any significant difference between patients treated with Lipofilling with respect to patients treated with ADSCs).

## 4. Discussion

SSc has an important social and emotional impact [30]. It is associated with increased functional impairment, body image distress due to skin lesions, and psychosocial comorbidity, particularly depression. Prevalence of depressive symptoms in SSc patients ranges from 36% to 65% and contributes to the worsening of any aspect of the disease [31].

Scleroderma patients report problems across multiple domains including fatigue, pain disability, sleep, interpersonal functioning, anxiety, and more generally physical and mental-health-related quality of life [30].

Fear of the disease (anxiety/panic) and depression are often not revealed by the patient because of the embarrassment of discovering an emotional illness. Scleroderma can be disfiguring and patients' psychosocial well-being is often affected more by disfigurement caused by facial changes. Low self-esteem alters social interactions and intimate relationships. The disease causes disability and may disrupt patients' ability to perform daily activities. Patients with mouth opening impairment cannot eat solid food, drink, and take care of their teeth thus highly limiting their social role in life.

Therapeutic strategies available today for chronic inflammatory diseases, such as SSc, often represent a way to obtain symptoms relief.

Au et al. [2] evaluated changes in vascular and musculoskeletal involvement in patients with interstitial lung disease comparing placebo treatment with oral cyclophosphamide (CYC). Authors demonstrated that there were no differences in dermal ulcer and musculoskeletal measures between the CYC and placebo groups at baseline and 24 months. Instead, mean oral aperture improved over time in the study participants.

Therapeutic repair encompasses the converging triad of rejuvenation, regeneration, and replacement strategies, which rely on self-healing processes, stem cell regeneration, and/or organ transplantation. Transplant medicine exploits the replacement strategy as a valuable option to recycle used parts and restore failing organ function by means of exogenous substitutes. It is, however, limited by donor shortage. Stem cell-based regeneration offers the next frontier of medical therapy through delivery of essentially unlimited pools of autologous progenitor cells to achieve structural and functional repair [32, 33].

However, translation into clinical applications requires the establishment of a regenerative medicine community of practice capable of bridging discovery with personalized treatment solutions. Indeed, this multidisciplinary specialized workforce will be capable of integrating the new science of embryology, immunology, and stem cell biology into bioinformatics and network medicine platforms, ensuring implementation of therapeutic repair strategies into individualized disease management algorithms.

Advanced cell-based therapies provide promising therapeutic possibilities to enhance repair or regeneration of damaged tissues also because their development may be greatly facilitated by the availability of an easily accessible and reproducible cell source.

In recent years, there has been growing emphasis on the use of mesenchymal stem cells (MSCs) for advanced cell therapy, due to their ability to be expanded in culture and to differentiate into multiple cell types. It is well established that MSCs secrete a broad spectrum of bioactive molecules with immunoregulatory and/or regenerative activities. Through direct cell-cell interaction or the secretion of various factors, MSCs can exert a great effect on local tissue repair by modulating the local environment and activating endogenous progenitor cells. Taken together, these properties make MSCs promising candidates for cell therapy in various diseases.

In particular, adipose-derived stem cells (ADSCs), isolated from stromal vascular fraction, are able to differentiate into various cell lineages such as chondrocytes, osteoblasts, and adipocytes and to exert potent immunomodulatory, proangiogenic, antiapoptotic, antifibrotic, and anti-inflammatory effects important in preventing tissue degeneration. In particular, ADSCs' angiogenic and immunomodulatory properties, including a suppressive response on collagen-reactive T cells and the capacity to restore immune tolerance by inhibiting the inflammatory response in vivo, strongly suggest their use for chronic pathologies, especially for autoimmune and inflammatory disorders [34].

Whilst a set of clinical trials are demonstrating safety and efficacy of personalized cell-based treatments using ADSCs, translation to patients is faced by an obstacle: the heterogeneity in 13 treatment methods. Standardizing and automating the manufacturing and characterization of ADSCs allows better comparisons and identification of optimal treatments. Moreover, cell-based techniques are costly, as cells need to be sent to centralized cell factories for cell expansion. Cell factories often are multipurpose (supporting numerous different therapies), utilize capital-intensive clean rooms and equipment, and need highly qualified personnel to implement manual processes. Transport of biological materials to and from centralized cell factories poses additional regulatory problems.

In fact, despite the advantages of cell-based approaches, in terms of both effectiveness and therapeutic potential, the diffusion of such therapies is still limited by the high costs of manufacturing processes and qualified personnel, as well as by the stringent rules that govern the isolation and expansion of cells for therapeutic use (GMP requirements).

Because of the above discussed limitations, it was necessary to turn back to another well-established technique exploiting ADSCs' potentialities as fat transplantation.

Over the years, autologous fat transplantation or “lipofilling technique” has become the first-choice procedure to fill depressed areas, restore anatomical saliences, and correct contour deformities or volumetric defects [3, 4].

Fat is ubiquitous and easily obtainable in large quantity with a minimal invasive collection procedure, limited patient discomfort, and minimal ethical considerations: it may be injected safely and efficaciously.

After harvesting fat, it must be processed in order to limit the blood or oil within the lipoaspirates so that only pure fat will be used for injection.

There are many different ways to process fat after its collection. Many authors described sedimentation by gravity or decantation, filtering, and centrifugation [35–38].

Currently, there is no agreement among authors in terms of which is the best method for processing fat grafts. In our study, we used decantation for a personal preference; in fact, we noticed that fat processed through decantation is more fluid and so more useful for scleroderma patients who present very fibrous areas, where the access and treatment are difficult. Many experimental studies designed to compare these 3 techniques were evaluated and the debate continues as to which is the best.

## 5. Conclusion

In this project, based on the successful results of our previous pilot study on cutaneous manifestations of systemic sclerosis (SSc) [39], we aim to compare two methods, ADSCs infiltration and fat transplantation, to evaluate their potential in treating diseases with few or no therapeutic choices. Such personalized therapies allow each patient to represent a resource for the treatment of his/her pathology.

ADSCs infiltration is an advanced method that is not performed by many laboratories in Italy. There are several intermediate and critical substeps required for effective cell manipulation and the entire process must comply with strict regulations. It is a procedure particularly useful in cases where not even the insertion of the smallest diameter cannula is possible, such as skin fibrosis. The main disadvantage is represented by its high costs due to cell preparation in specific laboratories and because it is performed in two separate sessions, thus increasing patient discomfort.

Autologous decanted fat transplantation allows us to obtain satisfactory results in terms of tissue trophism and mouth opening improvement, taking advantage of adipose-derived stromal cells properties and exploiting the fluidity of fat obtained from fat decantation especially to treat very fibrotic areas. Compared to the ADSCs injection, its main disadvantage is represented by cannula use, which is more traumatic with respect to thin needle employment. Cannula use also requires access sites, which are sutured possibly leading to scars. Fat adsorption is unpredictable and may result in irregularities and asymmetries with respect to more precise ADSCs injection. On the other hand, fat transplantation is a more economic procedure, requiring only one surgical step and one-day hospitalization.

In our study, we compared two techniques to determine whether one prevailed in terms of results and patient satisfaction. We noticed that both procedures obtained significant results but neither one emerged as a first-choice method.

We strongly believe in fat potentialities especially in treating immune-mediated chronic diseases as scleroderma and we hope to contribute to studies aimed at standardizing fat use. The present clinical experimentation should be regarded as a starting point for further experimental research and clinical trials.

## Figures and Tables

**Table 1:** Patients' characteristics: A: patients treated with ADSCs injection and L: patients treated with fat transplantation.

Patients	Age	Disease status	Disease duration	Localized scleroderma
1 A	23	1-year disease stabilization	3 years	Face and hands
2 A	25	9-year disease stabilization	5 years	Face
3 A	35	6-year disease stabilization	10 years	Face
4 A	38	6-year disease stabilization	10 years	Face
5 A	39	15-year disease stabilization	13 years	Face and hands
1 L	24	5-year disease stabilization	3 years	Face and hands
2 L	25	8-year disease stabilization	3 years	Face and hands
3 L	36	16-year disease stabilization	12 years	Face
4 L	39	8-year disease stabilization	15 years	Face
5 L	48	6-year disease stabilization	18 years	Face and hands

**Table 2:** Italian version of Mouth Handicap in Systemic Sclerosis Scale (IvMHISS) assesses the handicap with mouth disability in SSc. It consists of 12 items (each scored 0–4): 0 Never (Mai); 1 Rarely (Raramente); 2 Occasionally (Occasionalmente); 3 Often (Spesso); 4 Always (sempre).

1	I have difficulties opening my mouth Ho difficoltà ad aprire la bocca	0	1	2	3	4

2	I have to avoid certain drinks (sparkling, alcohol, etc.) Devo evitare alcuni tipi di bevande (frizzanti, alcoliche, ecc)	0	1	2	3	4

3	I have difficulties chewing Ho difficoltà a masticare	0	1	2	3	4

4	My dentist has difficulties taking care of my teeth Il mio dentist ha difficoltà a prendersi cura dei miei denti	0	1	2	3	4

5	My dentition had become altered La mia dentatura si è alterata	0	1	2	3	4

6	My lips are retracted and/or my checks are sunken Le mie labbra sono retratte e/o le mie guance sono infossate	0	1	2	3	4

7	My mouth is dry La mia bocca è secca	0	1	2	3	4

8	I have to drink often Devo bere spesso	0	1	2	3	4

9	My meals consist of what I can eat and not what I would like to eat Devo mangiare le cose che posso e non quelle che vorrei	0	1	2	3	4

10	I have difficulties speaking clearly Ho difficoltà a parlare con chiarezza	0	1	2	3	4

11	The appearance of my face is modified L'aspetto della mia faccia si è modificato	0	1	2	3	4

12	I have trouble with the way my face looks L'aspetto della mia faccia mi crea problemi	0	1	2	3	4

**Table 3:** Maximal Mouth Opening (MMO) by measuring the distance between the tips of upper and lower right incisive teeth.

Patients	Pretreatment (T0) opening mouth (cm)	Posttreatment opening mouth (cm)
1 L	3.6	4.2
2 L	3.4	3.8
3 L	2.6	3.4
4 L	3.1	3.8
5 L	3.3	4.2
1 A	3.4	4.4
2 A	2.5	3.1
3 A	3.3	3.7
4 A	2.9	3.6
5 A	3.2	4.0

**Table 4:** Visual Analogic Scale (VAS). Score ranging from 1 to 10, whereby 1 indicates no improvement and 10 the maximum possible improvement.

Patients	VAS
1 L	6
2 L	5
3 L	6
4 L	7
5 L	8
1 A	7
2 A	8
3 A	8
4 A	8
5 A	8

## References

[1] Poole J. L., MacIntyre N. J., deBoer H. N. (2013). Evidence-based management of hand and mouth disability in a woman living with diffuse systemic sclerosis (Scleroderma). *Physiotherapy Canada*.

[2] Au K., Mayes M. D., Maranian P. (2010). Course of dermal ulcers and musculoskeletal involvement in systemic sclerosis patients in the scleroderma lung study. *Arthritis Care & Research*.

[3] Coleman S. R. (2001). Structural fat grafts: the ideal filler?. *Clinics in Plastic Surgery*.

[4] Coleman S. R., Saboeiro A. P. (2007). Fat grafting to the breast revisited: safety and efficacy. *Plastic and Reconstructive Surgery*.

[5] Locke M., Windsor J., Dunbar P. R. (2009). Human adipose-derived stem cells: isolation, characterization and applications in surgery. *ANZ Journal of Surgery*.

[6] Fiorina P., Jurewicz M., Augello A. (2009). Immunomodulatory function of bone marrow-derived mesenchymal stem cells in experimental autoimmune type 1 diabetes. *Journal of Immunology*.

[7] Jurewicz M., Yang S., Augello A. (2010). Congenic mesenchymal stem cell therapy reverses hyperglycemia in experimental type 1 diabetes. *Diabetes*.

[8] Fioramonti P., Onesti M. G., Marchese C., Carella S., Ceccarelli S., Scuderi N. (2012). Autologous cultured melanocytes in vitiligo treatment comparison of two techniques to prepare the recipient site: erbium-doped yttrium aluminum garnet laser versus dermabrasion. *Dermatologic Surgery*.

[9] Masi A. T. (1980). Preliminary criteria for the classification of systemic sclerosis (scleroderma). Subcommittee for scleroderma criteria of the American Rheumatism Association Diagnostic and Therapeutic Criteria Committee. *Arthritis & Rheumatism*.

[10] Khanna D., Merkel P. A. (2007). Outcome measures in systemic sclerosis: an update on instruments and current research. *Current Rheumatology Reports*.

[11] Rodnan G. P., Lipinski E., Luksick J. (1979). Skin thickness and collagen content in progressive systemic sclerosis and localized scleroderma. *Arthritis and Rheumatism*.

[12] Dugar M., Woolford R., Ahern M. J., Smith M. D., Roberts-Thomson P. J. (2009). Use of electronic tonometer to assess skin hardness in systemic sclerosis: a pilot cross-sectional study. *Clinical and Experimental Rheumatology*.

[13] Enomoto D. N. H., Mekkes J. R., Bossuyt P. M. M., Hoekzema R., Bos J. D. (1996). Quantification of cutaneous sclerosis with a skin elasticity meter in patients with generalized scleroderma. *Journal of the American Academy of Dermatology*.

[14] Kissin E. Y., Schiller A. M., Gelbard R. B. (2006). Durometry for the assessment of skin disease in systemic sclerosis. *Arthritis Care and Research*.

[15] Falanga V., Bucalo B. (1993). Use of a durometer to assess skin hardness. *Journal of the American Academy of Dermatology*.

[16] Moon K. W., Song R., Kim J. H., Lee E. Y., Lee E. B., Song Y. W. (2012). The correlation between durometer score and modified Rodnan skin score in systemic sclerosis. *Rheumatology International*.

[17] Del Rosso A., Boldrini M., D'Agostino D. (2004). Health-related quality of life in systemic sclerosis as measured by the short form 36: relationship with clinical and biologic markers. *Arthritis Care and Research*.

[18] Poole J. L., Steen V. D. (1991). The use of the health assessment questionnaire (HAQ) to determine physical disability in systemic sclerosis. *Arthritis Care and Research*.

[19] Steen V. D., Medsger T. A. (1997). The value of the Health Assessment Questionnaire and special patient-generated scales to demonstrate change in systemic sclerosis patients over time. *Arthritis and Rheumatism*.

[20] Brower L. M., Poole J. L. (2004). Reliability and validity of the Duruöz Hand Index in persons with systemic sclerosis (scleroderma). *Arthritis & Rheumatism*.

[21] Sandqvist G., Eklund M. (2000). Validity of HAMIS: a test of hand mobility in scleroderma. *Arthritis Care and Research*.

[22] Wood R. E., Lee P. (1988). Analysis of the oral manifestations of systemic sclerosis (scleroderma). *Oral Surgery, Oral Medicine, Oral Pathology*.

[23] Marmary Y., Glaiss R., Pisanty S. (1981). Scleroderma: oral manifestations. *Oral Surgery, Oral Medicine, Oral Pathology*.

[24] Tanturri De Horatio C., Tirri E., Valletta R., Tirri G., Rengo S. (2000). Mouth opening in patients with systemic sclerosis: base analysis and during follow-up. *Minerva Stomatologica*.

[25] Bongi S. M., Del Rosso A., Miniati I. (2012). The Italian version of the Mouth Handicap in Systemic Sclerosis scale (MHISS) is valid, reliable and useful in assessing oral health-related quality of life (OHRQoL) in systemic sclerosis (SSc) patients. *Rheumatology International*.

[26] Nagy G., Kovács J., Zeher M., Czirják L. (1994). Analysis of the oral manifestations of systemic sclerosis. *Oral Surgery, Oral Medicine, Oral Pathology*.

[27] Albilia J. B., Lam D. K., Blanas N., Clokie C. M. L., Sándor G. K. B. (2007). Small mouths⋯big problems? A review of scleroderma and its oral health implications. *Journal of the Canadian Dental Association*.

[28] Dessy LA., Marcasciano M., Pacitti F., Rossi A., Mazzocchi M. (2015). A simple device for syringeto-syringe transfer during lipofilling. *Aesthetic Surgery Journal*.

[29] Scuderi N., Onesti M. G., Bistoni G. (2008). The clinical application of autologous bioengineered skin based on a hyaluronic acid scaffold. *Biomaterials*.

[30] Kwakkenbos L., Delisle V. C., Fox R. S. (2015). Psychosocial aspects of scleroderma. *Rheumatic Disease Clinics of North America*.

[31] Thombs B. D., Jewett L. R., Kwakkenbos L., Hudson M., Baron M. (2015). Major depression diagnoses among patients with systemic sclerosis: baseline and one month followup. *Arthritis Care & Research*.

[32] Alfano C., Chiummariello S., Fioramonti P., Innocenzi D., Scuderi N. (2006). Ultrastructural study of autologous cultivated conjunctival epithelium. *Ophthalmic Surgery, Lasers and Imaging*.

[33] Dessy L. A., Mazzocchi M., Corrias F., Ceccarelli S., Marchese C., Scuderi N. (2014). The use of cultured autologous oral epithelial cells for vaginoplasty in male-to-female transsexuals: a feasibility, safety, and advantageousness clinical pilot study. *Plastic and Reconstructive Surgery*.

[34] Maddali Bongi S., Del Rosso A., Mikhaylova S. (2015). District disability, fatigue and mood disorders as determinants of health related quality of life in patients with systemic sclerosis. *Joint Bone Spine*.

[35] Gimble J. M., Katz A. J., Bunnell B. A. (2007). Adipose-derived stem cells for regenerative medicine. *Circulation Research*.

[36] Minn K.-W., Min K.-H., Chang H., Kim S., Heo E.-J. (2010). Effects of fat preparation methods on the viabilities of autologous fat grafts. *Aesthetic Plastic Surgery*.

[37] Condé-Green A., de Amorim N. F. G., Pitanguy I. (2010). Influence of decantation, washing and centrifugation on adipocyte and mesenchymal stem cell content of aspirated adipose tissue: a comparative study. *Journal of Plastic, Reconstructive and Aesthetic Surgery*.

[38] Kurita M., Matsumoto D., Shigeura T. (2008). Influences of centrifugation on cells and tissues in liposuction aspirates: optimized centrifugation for lipotransfer and cell isolation. *Plastic and Reconstructive Surgery*.

[39] Scuderi N., Ceccarelli S., Onesti M. G. (2013). Human adipose-derived stromal cells for cell-based therapies in the treatment of systemic sclerosis. *Cell Transplantation*.

